# An X‐ray and γ‐ray combination strategy to exploit steep dose gradients for improved prostate cancer radiotherapy: A dosimetric comparison of Taichi Pro and Halcyon

**DOI:** 10.1002/acm2.70532

**Published:** 2026-02-27

**Authors:** Kuo Li, Yong Yin, Ting Zhu, Weipeng Sun, Shihao Wang, Zhenjiang Li

**Affiliations:** ^1^ Department of Radiation Oncology Physics & Technology Cancer Hospital of Shandong First Medical University Jinan China; ^2^ Our United Corp Xi'an China

**Keywords:** Halcyon, prostate cancer, rotating gamma system, Taichi Pro

## Abstract

**Purpose:**

This study aimed to evaluate a novel technological platform, Taichi Pro—which integrates a 6 MV flattening filterfree linear accelerator with a 18 source Rotating Gamma System (RGS) to generate steep dose gradients via multisource focused γ‐rays and noncoplanar arcs—for precision radiotherapy in prostate cancer. The work provides evidence to support the clinical adoption of hybridmodality radiotherapy devices.

**Methods:**

Fifteen prostate cancer patients were enrolled. For each patient, a dual‐modality Taichi Pro plan (RGS focused on the planning gross tumor volume (PGTV) + Linac covering the planning target volume (PTV)) and a Halcyon photon plan (three‐arc VMAT) were designed while maintaining clinically tolerable dose to organs at risk (OAR). Comparative assessments included planning target volume (PTV and PGTV) metrics (D_95%_, D_mean_, homogeneity index HI, conformity index CI), OAR doses (rectum V_40_/V_60_/D_2cc_, bladder V_40_, testis D_2cc_, etc.) and delivery efficiency to evaluate the ability to escalate target dose while sparing adjacent OARs.

**Results:**

All plans met institutional clinical constraints. Taichi Pro significantly increased PGTV D_mean_ (79.48 Gy ± 1.75 Gy) compared to Halcyon (73.25 Gy ± 0.55 24 Gy, *P* < 0.001) and D_max_ (122.74 Gy ± 8.69 Gy) compared to Halcyon (76.15 Gy ± 0.79 Gy, *P* < 0.001), albeit poorer homogeneity (HI: 0.50 ± 0.09 for Taichi Pro vs. 0.06 ± 0.01 for Halcyon, though within clinically acceptable limits). Taichi Pro significantly reduced rectum V_60_ (3.97% ± 3.25% vs. Halcyon 7.46% ± 4.78%, *P* = 0.016), and D_2cc_ (61.61 Gy ± 5.01 Gy vs. Halcyon 65.29 Gy ± 4.52 Gy, *P* = 0.040). Taichi Pro also significantly reduced testis D_2cc_ (2.39 ± 1.99 Gy) compared to (3.17 Gy ± 1.40 Gy, *P* = 0.006). Halcyon demonstrated significantly shorter beam‐on time (1.81 ± 0.23 minutes vs. 5.05 ± 1.59 minutes for Taichi Pro, *P* < 0.001).

**Conclusion:**

Utilizing the steep dose gradient characteristic of the RGS, the Taichi Pro dual‐modality system effectively achieved target dose escalation while simultaneously improving sparing of adjacent OARs. This approach holds the potential for enhancing patient treatment outcomes and quality of life.

## 1 INTRODUCTION

Prostate cancer represents the most prevalent malignancy and the second leading cause of cancer‐related mortality among men worldwide.^1^ Radiation therapy (RT) serves as a cornerstone curative intervention,^2^ offering an irreplaceable role in managing locally advanced disease and high‐risk presentations. Advances in precision radiotherapy—notably intensity‐modulated radiotherapy (IMRT)^3^ and volumetric modulated arc therapy (VMAT)—^4^have markedly enhanced target conformity through dynamic multi‐leaf collimator (MLC) modulation. Nevertheless, the inherent physical limitations of photon beams, including lateral scatter and a gradual dose fall‐off, coupled with the imperative to spare adjacent organs at risk (OARs), pose a central dilemma in prostate RT: how to simultaneously escalate target dose while rigorously protecting OARs remains a pivotal optimization challenge. Evidence suggests that dose escalation to intraprostatic dominant lesions via simultaneous integrated boost IMRT up to 95 Gy can improve the 5‐year biochemical control rate by 7% without increasing ≥Grade 3 toxicities.^5^ Conversely, the risk of RT‐related toxicity to OARs—such as the rectum,^6^ bladder,^7^ and bowel—^8^rises significantly with higher dose and volume exposure.

Conventional photon‐based radiotherapy systems, such as Halcyon, deliver efficient treatments through VMAT. Yet their dose gradient index (GI) typically ranges from 4.0 to 5.0,^9^ which may be insufficient to achieve the rapid dose fall‐off required to meet the stringent OAR dose‐volume constraints mandated in modern prostate radiotherapy protocols.^10^ In recent years, hybrid radiotherapy systems have attracted growing interest due to the complementary physical properties of their constituent technologies. The Taichi Pro system exemplifies such innovation by integrating a 6 MV flattening‐filter‐free (FFF) linear accelerator with an 18‐source Rotating Gamma System (RGS) on a single gantry. This design merges the broad‐field modulation capability of the Linac with the characteristically steep dose gradients of the RGS. The RGS achieves its sharp dose fall‐off through multi‐source focusing and a non‐coplanar arc arrangement, generating a high‐gradient dose distribution around the target. Compared to conventional photon‐based plans, the volume enclosed by the 50% isodose line is reduced by 30–40%.^11^, ^12^ Meanwhile, the Linac employs dynamic MLC technology to optimize dose uniformity within the central target region.^3^

The RGS utilizes multiple Co‐60 sources that converge at the isocenter through mechanically collimated channels.^13^ This configuration produces exceptionally steep dose gradients by means of multi‐source focusing and non‐coplanar arc delivery. Within the Taichi Pro platform, the integration of the RGS with a 6 MV FFF linac was designed to harness the RGS's sharp dose fall‐off for focal escalation to the planning gross tumor volume (PGTV), while employing the linac's broad‐field modulation capability to achieve comprehensive elective coverage. This dual‐modality approach seeks to transcend the conventional trade‐off between target dose escalation and OAR sparing in anatomically confined regions such as the prostate.

This study focuses on prostate cancer, systematically comparing the dosimetric performance of Taichi Pro dual‐modality plans with Halcyon photon‐only plans. The aim was to evaluate a novel technological option for precision radiotherapy in prostate cancer and to provide evidence‐based support for the clinical adoption of hybrid‐modality radiotherapy devices.

## 2 MATERIALS AND METHODS

### 2.1 Target and prescription definition

Fifteen prostate cancer patients who received VMAT at our institution between January 2022 and October 2024 were randomly selected. Target delineation followed RTOG consensus guidelines.^14^, ^15^ The gross tumor volume (GTV) encompassed the entire prostate gland and its capsular region. A uniform 3 mm expansion from the GTV generated the PGTV. The clinical target volume (CTV) included the pelvic lymphatic drainage regions and the prostateseminal vesicle region. The planning target volume (PTV) was created by uniformly expanding the CTV by 5 mm, except for a 3 mm expansion posteriorly toward the rectum. The bladder, rectum, femoral heads, bowel bag, and other normal structures were delineated as OARs. The PTV served as the primary planning target, while the PGTV was the boost target. The mean PGTV volume was 121.00  ±  46.36 cc, and the PTV volume was 861.57  ±  176.74 cc. The prescription dose was 70 Gy to the PGTV and 50.4 Gy to the PTV, delivered in 28 fractions at 2.5 Gy and 1.8 Gy per fraction, respectively. All contours were drawn by a single physician.

### 2.2 Accelerator

The Halcyon accelerator (Varian Medical Systems, Palo Alto, CA, USA) features an Oring structure and delivers a 6 MV FFF beam at a maximum dose rate of 800 MU/min. It was equipped with a duallayer MLC operating in SX1 mode: the proximal bank has 58 leaves and the distal bank 56 leaves, all with a projected width of 1.0 cm at isocenter. The stacked, staggered leaf design eliminates conventional jaws, yielding a leaf transmission of 0.1‰. Each leaf traverses the full 28 cm field width and has a 23.4 cm radius of curvature with a 7.7 cm height.

**FIGURE 1:**
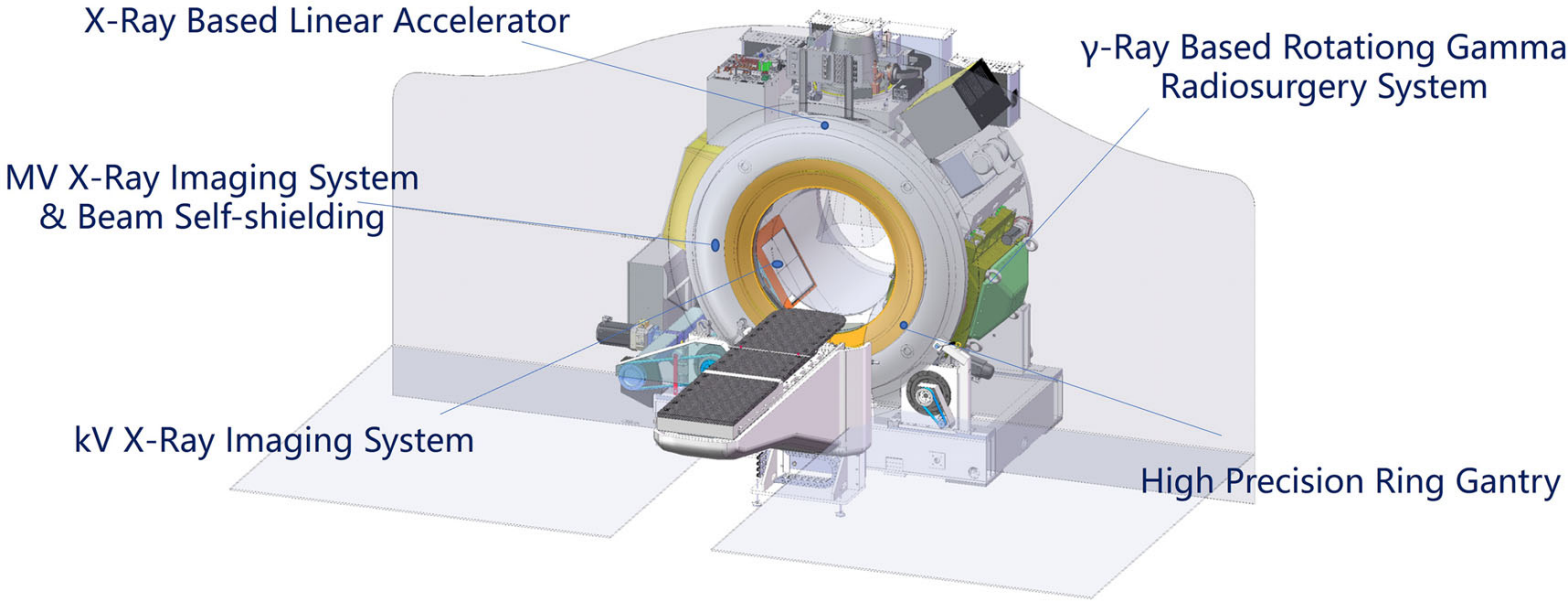
Schematic views of the TAICHI platform.

The Taichi Pro dual‐modality system (OUR United Corp., Xi'an, China, Figure 1) integrates a linear accelerator with a RGS within a single slip‐ring gantry. The gantry supports continuous rotation with a variable speed ranging from 0 to 1 revolution per minute, enabling modulation of dose delivery per angle during arc therapy. The Linac component operates at a 6 MV FFF energy mode, delivering a peak dose rate of 14 Gy/min and providing a maximum field size of 40 × 40 cm. It was equipped with a set of primary collimating jaws for initial field shaping, in addition to a dynamic MLC that was functionally similar to those used in conventional C‐arm linear accelerators. It features a central leaf width of 5 mm and peripheral leaf widths of 10 mm. The RGS incorporates 18 Co‐60 sources focused at the isocenter, delivering a dose rate of 3.5 Gy/min and was equipped with adjustable collimators. Seven collimator sizes were available for each source, with diameters of 6, 9, 12, 16, 20, 25, and 35 mm. This unit delivers radiation via 18 non‐coplanar arcs through gantry rotation. An on‐board kilovoltage image guidance system was integrated into the gantry for image‐guided patient positioning. The system implements a unified drive mechanism ensuring that all three core modules (X‐ray, γ‐ray and real‐time imaging system) share the same isocenter.

### 2.3 Treatment planning

All plans were designed by the same experienced medical physicist. Halcyon plans were created using the Eclipse treatment planning system (version 16.1, Varian Medical Systems, Palo Alto, CA). Plans for the Halcyon accelerator utilizing the 6‐MV FFF beam mode consisted of three coplanar full‐arc VMAT fields, with the collimator rotated to angles between 10° to 30° to reduce interleaf leakage dose. The Photon Optimizer (PO, v16.1) and Anisotropic Analytical Algorithm (AAA, v16.1) were employed for optimization and dose calculation, respectively. The Taichi plan was designed using the RT Pro TPS (V2.5.8.4914, OUR United Corp., Xi'an, China), which was a combination of the work done by Redpath A T^16^ and Mohan R.^17^ For the RGS of Taichi, each shot employed a radioactive Co‐60 source delivering rotational multi‐focused arcs. The PGTV plan was optimized using the Co‐60 γ‐ray beam model, with dose calculation performed by the Fast Photon algorithm. Planning incorporated two or more collimator sizes (from the available set of 7) selected according to the target volume size and shape. After achieving a dose distribution that delivered 50% of the prescription dose to the PGTV, a 6 MV FFF photon plan comprising three coplanar full arcs was optimized based on this distribution using the Collapsed Cone Convolution (CCC) algorithm, yielding the final dualmodality plan. Optimization objectives for PTV coverage and OAR sparing were kept as consistent as possible between the two planning systems, adhering to institutional clinical protocols.

### 2.4 Plan evaluation

The dose volume histograms (DVHs) of the target and OARs were exported from the two plans of the planning systems. For the dosimetric evaluation, the following indices were calculated: the maximum and minimum doses, represented by the doses received by 2% (D_2_) and 98% (D_98_) of the target volume, respectively. In addition, the homogeneity index (HI) and conformity index (CI) of the target were calculated as follows:

$$HI=\frac{D_{2}-D_{98}}{D_{press}}$$

and

$$CI=\frac{T{V_{PIV}}^{2}}{TV\times TV_{RX}}$$

In the above equation, D_press_ represents the prescription dose; TV_PIV_ represents the volume of the target volume overlapping with the prescription dose; TV represents the target volume; TV_RX_ represents the volume covered by the prescription dose. For the OARs, 2cc volume receiving the highest dose for the rectum (D_2cc_) of the rectum, penile bulb, testis and small intestine were reported. The mean dose (D_mean_) and the volumes of the bladder, rectum and femur‐head (L/R) receiving more than 40, 50 or 60 Gy (V_40_, V_50_ or V_60_) were reported. The total delivery time of all the plans was counted. In this study, the dose constraints for OARs were referred to in Table 1. Certain dose parameter (like D_2cc_ of Rectum) was kept as low as possible due to overlap with the PGTV.

**TABLE 1:** The clinical objectives of organs at risk.

	Desirable dose		Acceptable dose
OAR	Specification	Dose (Gy)	Dose (Gy)
Bladder	V_40_	40%	60%
Rectum	V_40_	50%	65%
	V_60_	40%	60%
	D_2cc_	low	–
Femur‐Head L	V_40_	10%	40%
	V_50_	5%	10%
Femur‐Head R	V_40_	10%	40%
	V_50_	5%	10%
penile bulb	D_2cc_	40 Gy	42 Gy
testis	D_2cc_	4 Gy	6 Gy
small intestine	D_2cc_	52 Gy	55 Gy

### 2.5 Statistical analysis

Any differences among the thirty plans (from fifteen patients) according to the Halcyon and Taichi Pro systems were analyzed by SPSS 27.0 (SPSS Inc., Chicago, IL, USA). Non‐parametric statistical analysis was performed using the Mann‐Whitney U test to assess differences between the Halcyon and Taichi Pro, where *P *< 0.05 was considered statistically significant.

## 3 RESULTS

**FIGURE 2:**
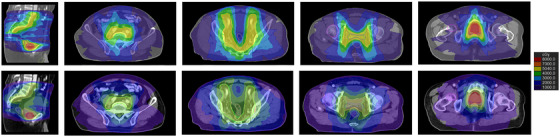
The dose distribution of Taichi Pro (1st line) and Halcyon (2nd line) plans.

All prostate cancer plans designed by the Halcyon and Taichi Pro accelerators met the clinical requirements regarding the PGTV/PTV coverage and radiation dose of OARs, as shown in Figure 2.

### 3.1 PTV coverage

Results for target coverage were summarized in Table 2 and Supplementary Fig. (a), (b). For the volume covering 95% of the target, the Taichi Pro system delivered dose values significantly closer to the prescription dose with less excessive dose deposited outside the target volume. Statistically significant differences in prescription dose coverage were observed for both PGTV and PTV (*P* < 0.001 and *P* = 0.001, respectively). However, no significant differences in CI values were found between the two plans. Compared with the Halcyon system, the Taichi Pro system significantly increased the D_mean_ (79.48 Gy ± 1.75 Gy vs. 73.25 Gy ± 0.55 Gy, *P* < 0.001) and D_max_ (122.74 Gy ± 8.69 Gy vs. 76.15 Gy ± 0.79 Gy, *P* < 0.001) to PGTV. This improvement, however, was achieved at the expense of compromised HI (0.50 ± 0.09 vs. 0.06 ± 0.01, *P* < 0.001). Therefore, the dose distributions of the two systems for a typical patient in this study were indicated in Figure 2.

**TABLE 2:** Dose of the planning target volume index of two plans for prostate cancer plans.

Dose index		PGTV	PTV	*P*1 value	*P*2 value
D_95%_(Gy)	Halcyon	71.32 ± 0.47	50.94 ± 0.36	< 0.001	0.001
	Taichi Pro	70.32 ± 0.43	50.59 ± 0.10		
D_mean_(Gy)	Halcyon	73.25 ± 0.55	53.82 ± 1.14	< 0.001	0.245
	Taichi Pro	79.48 ± 1.75	53.37 ± 0.72		
D_max_(Gy)	Halcyon	76.15 ± 0.79	–	< 0.001	–
	Taichi Pro	122.74 ± 8.69	–		
HI	Halcyon	0.06 ± 0.01	0.33 ± 0.14	< 0.001	0.934
	Taichi Pro	0.50 ± 0.09	0.34 ± 0.13		
CI	Halcyon	0.89 ± 0.03	0.70 ± 0.07	0.184	0.590
	Taichi Pro	0.87 ± 0.02	0.69 ± 0.05		

^#^ *P*1 value = Halcyon plans vs Taichi Pro plans for PGTV; *P*2 value = Halcyon plans vs Taichi Pro plans for PTV.

^#^Data were mean  ±  SD (*n* = 15).

### 3.2 Dose of OAR

**FIGURE 3:**
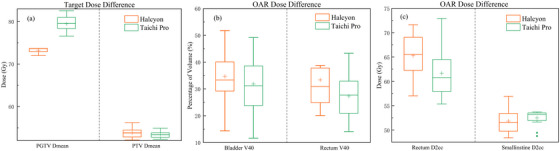
Dosimetric comparison for target region and OARs between Halcyon plan and TaiChi Pro plan. Target dose (a) and OAR dose constraints comparisons (b and c), plotted as the difference between the Halcyon and TaiChi Pro plans.

Regarding OARs (Table 3, Figure 3(b and c) and Supplementary Fig. (c)–(i)), no significant differences were observed in the high‐dose values (D_2cc_) for the penile bulb and small bowel between the two systems (*P* > 0.05). Due to their proximity to the PGTV and its high‐dose region, the bladder and rectum received higher doses. Taichi Pro reduced the bladder V_40_ to 31.75% ± 10.23%, though this was not statistically significant (*P* = 0.431). In contrast, Taichi Pro demonstrated superior rectum sparing compared to Halcyon, achieving significant reductions in V_60_ (by 3.49%, *P* = 0.016). Concurrently, the high‐dose parameter D_2cc_ for the rectum decreased significantly from 65.29 Gy ± 4.52 Gy with Halcyon to 61.61 Gy ± 5.01 Gy with Taichi Pro. For male patients, the testes were highly radiosensitive organs with strict dose constraints. Although Halcyon provided good testicular dose sparing in prostate cancer treatment (3.17 Gy ± 1.40 Gy), Taichi Pro achieved further significant reduction in testicular dose (2.39 Gy ± 1.99 Gy), with a statistically significant difference (*P* = 0.006).

**TABLE 3:** Dose of the organs at risk index of two plans for prostate cancer plans.

OAR	Dose index	Halcyon	Taichi Pro	P value
Bladder(Gy)	V_40_	34.67 ± 10.51	31.75 ± 10.23	0.431
Rectum(Gy)	V_40_	33.39 ± 13.18	27.23 ± 7.01	0.152
	V_60_	7.46 ± 4.78	3.97 ± 3.25	0.016
	D_2cc_	65.29 ± 4.52	61.61 ± 5.01	0.040
Femur‐Head L(Gy)	V_40_	0.47 ± 0.87	0.06 ± 0.15	0.263
	V_50_	0.00 ± 0.00	0.00 ± 0.00	1.000
Femur‐Head R(Gy)	V_40_	0.88 ± 1.47	0.18 ± 0.53	0.052
	V_50_	0.00 ± 0.00	0.00 ± 0.00	1.000
Penile bulb(Gy)	D_2cc_	28.05 ± 10.13	23.49 ± 9.36	0.491
Testis(Gy)	D_2cc_	3.17 ± 1.40	2.39 ± 1.99	0.006
Small Intestine(Gy)	D_2cc_	51.83 ± 2.39	52.52 ± 1.50	0.217

^#^Data were mean  ±  SD (*n* = 15).

### 3.3 Execution efficiency

The time metrics for plan generation and treatment delivery were summarized in Table 4. The planning time for the Taichi Pro dual‐modality workflow (44.69 ± 2.43 minutes) was significantly shorter compared to that for Halcyon VMAT plans (52.41 ± 4.20 minutes, *P* < 0.001). Conversely, the beam‐on delivery time per fraction was significantly shorter for Halcyon (1.81 ± 0.23 minutes) than for Taichi Pro (6.11 ± 1.63 minutes, *P* < 0.001).

**TABLE 4:** MU and delivery time of two plans for prostate cancer plans.

		Taichi Pro		
Index	Halcyon	γ‐ray	X‐ray	*P* value
Planning time (min)	52.41 ± 4.19	7.30 ± 1.19	37.39 ± 1.91	< 0.001
MU	965.19 ± 122.11	–	1552.33 ± 217.65	< 0.001
Delivery time (min)	1.81 ± 0.23	3.15 ± 0.23	3.21 ± 1.37	< 0.001

^#^ Data were mean  ±  SD (*n* = 15).

To assess the deliverability of the planned dose distributions, quality assurance (QA) was performed for both systems using a 3 mm distance‐to‐agreement and 2% dose difference (3 mm/2%) gamma analysis criterion. The mean gamma passing rates were 98.57% ± 1.25% for Halcyon plans and 96.72% ± 1.26% for Taichi Pro X‐ray plans, with the difference being statistically significant (*P* < 0.01). Both systems achieved passing rates well above the commonly adopted clinical action level of 95%.

## 4 DISCUSSION

The Taichi Pro system has been validated as capable of producing clinically acceptable, high‐quality treatment plans. Distinct from conventional single‐modality accelerators, it integrates a linac and an RGS at a common isocenter, forming a dual‐energy delivery platform with continuous gantry rotation enabled by a slip‐ring design. The strategic application in this study differs from previous implementations in other disease sites. For instance, earlier work in lung SBRT primarily utilized the RGS for dose delivery, supplemented by the linac for homogenization, resulting in hybrid plans comparable to those from RGS alone.^18^ In contrast, the present prostate cancer study employs a dedicated complementary workflow: the steep gradient of the RGS was strategically assigned to deliver a focal boost exclusively to the PGTV, while the broad‐field modulation capability of the linac covers the larger elective PTV. This “RGS‐for‐escalation + Linac‐for‐coverage” paradigm was designed to leverage the distinct physical advantages of each modality to address the dual clinical needs in prostate cancer—maximizing dose to the gross disease while safely treating the subclinical region. Furthermore, whereas a prior study in pancreatic cancer emphasized theoretical benefits through bio‐modeling,^19^ our analysis provides a direct, quantitative dosimetric comparison against a contemporary clinical standard, offering tangible evidence of this strategy's potential to improve the therapeutic ratio. Thus, the novelty of this work lies in validating this site‐specific hybrid approach, which demonstrates the feasibility of achieving significant focal dose escalation alongside enhanced OAR sparing—a clear advance over advanced photon‐only techniques in this anatomically constrained site.

The steep dose gradient achieved by Taichi Pro originates from the distinct penumbra characteristics of its RGS component compared to the 6 MV FFF beam of Halcyon. While a single Co‐60 source exhibits a broader geometric penumbra due to its larger physical size, the RGS utilizes multi‐source focusing.^13^, ^20^ The superposition of dose from 18 individually collimated sources, delivered through non‐coplanar arcs, produces an exceptionally sharp effective penumbra at the target periphery. In contrast, the penumbra of a 6 MV FFF beam was fundamentally limited by lateral electron scatter and finite source size, even when paired with a high‐resolution MLC. This fundamental physical distinction underpins the RGS's superior capability to confine high doses and create a rapid dose fall‐off—a characteristic directly leveraged in our “RGS‐for‐escalation” strategy to spare adjacent OAR.

This study conducted a comprehensive comparative analysis of the Taichi Pro radiotherapy system versus the Halcyon system for prostate cancer radiotherapy. The results demonstrate significant advantages for the Taichi Pro system in terms of target dose escalation and sparing of OARs. Regarding target dose coverage, Taichi Pro achieved superior values for PGTV D_95%_, D_mean_, and D_max_ compared to Halcyon, indicating its enhanced ability to concentrate high‐dose radiation within the target volume and deliver dose more precisely. This effect was primarily attributable to its Co‐60 γ‐ray RGS, which generates a steep dose gradient around the target through multi‐source focusing and non‐coplanar arc delivery.^20^, ^21^ While this comes at the cost of some intra‐target homogeneity, it creates a sharper dose fall‐off at the target periphery, forming the physical basis for improved protection of surrounding OARs. Reducing the dose and irradiated volume to OARs was crucial for minimizing radiotherapy‐related toxicities, such as rectum bleeding,^22^ cystitis,^23^ and sexual dysfunction.^24^ A key finding of this study was the pronounced advantage of Taichi Pro in rectum sparing. Compared to Halcyon, Taichi Pro significantly reduced rectum V_40_, V_60_, and the high‐dose parameter D_2cc_. These reductions hold considerable clinical potential, likely translating into improved patient quality of life and treatment tolerance. Furthermore, Taichi Pro significantly lowered the dose to the testes, which was important for preserving fertility and minimizing gonadal dysfunction. The improvement in rectum sparing may be related to the inherently non‐coplanar beam paths of the RGS, which can avoid portions of the rectum or optimize beam angles. The reduction in testicular dose may be attributed to the more focused beam profile of the RGS and/or the optimized beam arrangement from the Linac component, thereby minimizing scatter dose to the lower pelvic region.

The significantly higher HI observed in Taichi Pro plans was an inherent characteristic of the RGS component. The multi‐source focusing technique produces an extremely steep dose gradient, which was highly effective for sparing OARs but inevitably leads to a more heterogeneous dose distribution within the target, including the presence of high‐dose hotspots. This represents a well‐established trade‐off in radiosurgery and focused radiotherapy techniques,^25^ where a steeper gradient was achieved at the expense of intra‐target dose homogeneity. While the RGS produces a steep dose gradient, the resulting hotspots within the PTV can frequently exceed 200% of the prescribed dose,^26^, ^27^ which may elevate the D_2cc_ in overlapping OARs. GTV dose escalation has been associated with improved local control in prostate cancer. Prior studies^28^, ^29^, ^30^ using stereotactic techniques have shown favorable biochemical control without significant increase in late genitourinary toxicity. Regarding urethral risk, the “bull's‐eye” distribution of RGS inherently places hotspots away from the urethra. Nonetheless, we acknowledge the need for longer follow‐up and larger cohorts to fully validate the safety of this approach.

To mitigate such risks, this study employed strategies including mixed collimator sizes, sector blocking, and daily CBCT‐guided adaptive radiotherapy. Furthermore, as the dose fall‐off becomes steeper, even minor displacements of the target or OARs could lead to under‐dosage of the PGTV or over‐dosage of adjacent critical structures. Intrafraction prostate motion was a critical concern for prolonged treatments with steep dose gradients. While daily CBCT corrects inter‐fraction errors, the Taichi Pro platform was equipped with real‐time kilovoltage imaging, enabling continuous intrafraction guidance throughout delivery. Moreover, to further enhance motion management, we can further integrate fiducial marker tracking and optical surface monitoring systems. This integrated approach will provide redundant real‐time position feedback. This multi‐layered strategy was well suited to the dosimetric precision required by the RGS and the extended treatment time of the hybrid modality, ensuring that focal dose escalation was delivered accurately without compromising OAR sparing.

A notable finding was the significantly shorter planning time for Taichi Pro compared to Halcyon. This was likely attributable to the distinct workflow of its dual‐modality strategy: the RGS plan for the PGTV boost typically employs a limited set of standard collimators and non‐coplanar arcs, which can be optimized efficiently, while the complementary Linac plan for the PTV coverage constitutes a conventional VMAT optimization. In contrast, achieving stringent OAR sparing with a photon‐only VMAT plan for prostate cancer often requires more iterative optimization. However, this planning efficiency was offset by a significantly longer delivery time for Taichi Pro. This was primarily due to the sequential delivery of the RGS arcs and Linac arcs, which includes the time overhead for gantry rotation and modality switching. Consequently, the total estimated time per clinical fraction still favors Halcyon. The longer treatment time for Taichi Pro remains a practical consideration, as it may affect patient comfort and intra‐fraction motion management.

The QA results further support the clinical feasibility of the proposed hybrid strategy. Gamma‐analysis specifically validates the accurate deliverability of the complex modulation from the Linac component within the integrated plan. The high passing rate (> 96%) confirms that the Linac fields can be delivered with excellent clinical precision. Unlike linac X‑ray beams, which were generated pulse‑by‑pulse via electron impact, the Co‑60 sources in the RGS were fixed radioactive sources with a predictable, time‑decaying output (half‑life 5.27 years). Their dose delivery was pre‑calibrated and plan‑independent, determined solely by source geometry, collimator size, and gantry rotation. Thus, patient‑specific QA was not clinically required; accuracy was ensured by periodic constancy checks.^13^, ^20^ In this study, the Linac component passed patient‐specific QA, while the RGS component adhered to its established periodic QA protocol. Together, these complementary verification processes confirm that the composite steep‐gradient dose distribution of the Taichi Pro dual‐modality plan was clinically deliverable with high overall precision.

## 5 CONCLUSION

In summary, the Taichi Pro hybrid radiotherapy system demonstrates considerable potential for prostate cancer treatment. It effectively enables target dose escalation while simultaneously improving the protection of adjacent OARs, potentially leading to enhanced therapeutic outcomes and quality of life for patients. Future technological refinements and accumulated clinical experience may foster broader adoption of Taichi Pro in prostate radiotherapy, further advancing the field.

## AUTHOR CONTRIBUTIONS

**Kuo Li** and **Shihao Wang** contributed to data collection and analysis and drafted the initial manuscript. **Kuo Li** also performed statistical analysis and figure creation. **Kuo Li** and **Yong Yin** worked together to complete the manuscript revision and experimental protocol design. **Zhenjiang Li** overseeing study design, research methodology, and funding acquisition. **Ting Zhu** and **Weipeng Sun** were involved in data analysis and provide technical support. All other authors contributed to data collection. All authors reviewed the results and approved the final version of the manuscript.

## CONFLICT OF INTEREST STATEMENT

The authors declare that they have no known competing financial interests or personal relationships that could have appeared to influence the work reported in this paper.

## ETHICAL APPROVAL STATEMENT

This study protocol has been approved by the Ethics Committee of the Affiliated Cancer Hospital of Shandong First Medical University (approval number: SDTHEC202508024).

## Supporting information



Supporting Information

## Data Availability

The datasets used and/or analyzed during the current study were available from the corresponding author on reasonable request.
